# Risk assessment model for first-cycle chemotherapy-induced neutropenia in patients with solid tumours

**DOI:** 10.1111/j.1365-2354.2009.01121.x

**Published:** 2010-09

**Authors:** A LÓPEZ-POUSA, J RIFÀ, A CASAS DE TEJERINA, JL GONZÁLEZ-LARRIBA, C IGLESIAS, JA GASQUET, A CARRATO

**Affiliations:** 1 Medical Oncology Department, Santa Creu i Sant Pau HospitalBarcelona; 2 Medical Oncology Department, Son Dureta HospitalPalma de Mallorca; 3 Medical Oncology Department, Virgen del Rocío HospitalSevilla; 4 Medical Oncology Department, Clínico San Carlos HospitalMadrid; 5 Medical Department, Salutis Research S. A.Barcelona; 6 Medical Department, AMGEN S.A.Barcelona; 7 Medical Oncology Department, Elche University HospitalElche, Spain

**Keywords:** solid tumours, neutropenia, predictive model

## Abstract

LÓPEZ-POUSA A., RIFÀ J., CASAS DE TEJERINA A., GONZÁLEZ-LARRIBA J.L., IGLESIAS C., GASQUET J.A. & CARRATO A. (2010) *European Journal of Cancer Care* **Risk assessment model for first-cycle chemotherapy-induced neutropenia in patients with solid tumours**

Chemotherapy-induced neutropenia, the major dose-limiting toxicity of chemotherapy, is directly associated with concomitant morbidity, mortality and health-care costs. The use of prophylactic granulocyte colony-stimulating factors may reduce the incidence and duration of chemotherapy-induced neutropenia, and is recommended in high-risk patients. The objective of this study was to develop a model to predict first-cycle chemotherapy-induced neutropenia (defined as neutropenia grade ≥3, with or without body temperature ≥38°C) in patients with solid tumours. A total of 1194 patients [56% women; mean age 58 ± 12 years; 94% Eastern Cooperative Oncology Group (ECOG) status ≤1] with solid tumours were included in a multi-centre non-interventional prospective cohort study. A predictive logistic regression model was developed. Several factors were found to influence chemotherapy-induced neutropenia. Higher ECOG status values increased toxicity (ECOG 2 vs. 0, *P*= 0.003; odds ratio 3.12), whereas baseline lymphocyte (*P*= 0.011; odds ratio 0.67) and neutrophil counts (*P*= 0.026; odds ratio 0.90) were inversely related to neutropenia occurrence. Sex and treatment intention also significantly influenced chemotherapy-induced neutropenia (*P*= 0.012). The sensitivity and specificity of the model were 63% and 67% respectively, and the positive and negative predictive values were 17% and 94% respectively. Once validated, this model should be a useful tool for clinical decision making.

## INTRODUCTION

Patients receiving myelosuppressive chemotherapy frequently develop severe neutropenia or febrile neutropenia. Myelosuppressive chemotherapy regimens are regularly used to treat a wide variety of malignancies, and chemotherapy-induced neutropenia is one of the major dose-limiting toxicities seen in clinical oncology practice. Neutropenia, and more specifically febrile neutropenia, are clinically relevant issues with a negative impact on quality of life, causing increased morbidity and mortality rates, and elevating treatment costs (Schimpff *et al.* 1978; Pizzo *et al.* 1986; Talcott *et al.* 1992; Hughes *et al.* 2002). In future, chemotherapy-induced neutropenia will become an even greater issue as elderly populations increase in developed countries, leading to a higher prevalence of cancers and an increase in the age-related risk of chemotherapy-induced neutropenia (Lyman *et al.* 2003).

The reported incidence and prevalence of neutropenia vary widely. One of the most reliable estimates cited in the literature suggests an incidence of 7.83 neutropenic hospitalisations per 1000 cancer patients (Caggiano *et al.* 2005). However, neutropenia has been observed in 6–50% of patients, depending on the cancer type, disease staging, patient functional status and chemotherapy regimen (Smith *et al.* 2006). In the USA, reported inpatient mortality rates associated with grades 3 and 4 neutropenia range from 3.4% to 10.5%, with an overall mortality ranging from 6.8% to 9.5% (Crawford *et al.* 2004; Caggiano *et al.* 2005; Kuderer *et al.* 2006).

Toxicity during the first chemotherapy cycle is an important indicator of future neutropenic events, tolerance to chemotherapy and overall disease response. Several studies have shown that patients with poor chemotherapy tolerance and associated complications during the first cycle ultimately have a worse clinical outcome than their counterparts without such adverse responses (Blay *et al.* 1996; Crawford *et al.* 2004). This variability is probably related to modifications of planned chemotherapy treatments, such as decreasing the chemotherapy dose, increasing the interval between cycles, changing chemotherapeutic agents and possibly even stopping treatment completely. These alterations usually diminish the response rate and the patient's overall survival (Dixon *et al.* 1986; Kwak *et al.* 1990; Lepage *et al.* 1993; Bonadonna *et al.* 1995; Chrischilles *et al.* 2003; Lyman & Delgado 2003).

For several years, physicians have used prophylactic granulocyte colony-stimulating factors to reduce the incidence and duration of chemotherapy-induced neutropenia/febrile neutropenia, thus improving the patient's quality of life (Fazio & Glaspy 1991; Padilla & Ropka 2005) and reducing the rate of associated complications, such as prolonged hospitalisation (Vogel *et al.* 2005) and empiric use of broad-spectrum antibiotics. However, granulocyte colony-stimulating factors treatment is not administered to all patients at high risk of neutropenia receiving chemotherapy because of its cost and a paucity of conclusive studies for some diseases and chemotherapy regimens. Nevertheless, the guidelines of the American Society of Clinical Oncology and of the European Organization for Research and Treatment of Cancer, which are based on the best evidence currently available, recommend the use of granulocyte colony-stimulating factors primary prophylaxis when the overall risk of febrile neutropenia from chemotherapy and patient-related factors (e.g. age, advanced disease) is ≥20% (Aapro *et al.* 2006; Smith *et al.* 2006). However, despite these recommendations, both sets of guidelines recognise the need for better tools to improve the risk assessment of chemotherapy-induced neutropenia.

Since the early years of systemic chemotherapy, efforts have been made to create a model or tool for predicting chemotherapy-induced neutropenia that would allow the stratification of patients and treatments according to risk profiles. The literature contains numerous studies and small case series that have led to the development of such models for specific neoplasias (e.g. non-Hodgkin's lymphoma, breast cancer, lung cancer) or chemotherapy regimens (Blay *et al.* 1996; Silber *et al.* 1998; Klastersky *et al.* 2000; Lyman 2000; Ray-Coquard *et al.* 2003; Savvides *et al.* 2003). One of the most comprehensive reviews of these risk-model studies identified common factors of the 14 models proposed for chemotherapy-induced neutropenia, including age, performance status, nutritional status, chemotherapy dose intensity and baseline blood cell counts. Unfortunately, conclusive results were limited because of the different study designs, small patient numbers and diverse chemotherapy regimens used in these studies (Silber *et al.* 1998; Aslani *et al.* 2000; Intragumtornchai *et al.* 2000; Voog *et al.* 2000; Savvides *et al.* 2003). The development of a risk assessment model for chemotherapy-induced neutropenia in patients with solid tumours remains a priority. In this article, we present our preliminary results in developing such a model, based on data from the DELFOS study.

## METHODS

### Study design

The DELFOS study was a multi-centre, prospective, non-interventional cohort study undertaken in Spain to assess the haematological toxicity risk in patients with solid tumours during their first three chemotherapy cycles. The chemotherapy regimen administered to each patient was determined on the basis of normal clinical practice.

Patients were included in the study over a 10-month period from 15 March 2004 to 5 January 2005. Patient data were recorded before each of their first three chemotherapy cycles, after the third chemotherapy cycle, and one year after chemotherapy initiation. All data were verified by qualified personnel.

The study was approved by the ethical review boards of all participating centres and was sponsored by the Spanish Society of Medical Oncology. All patients gave their written informed consent.

### Inclusion criteria

Eligible patients were adults (aged ≥18 years) with a histologically confirmed solid tumour who had not previously received chemotherapy, with an Eastern Cooperative Oncology Group (ECOG) performance status score of ≤2, and a good bone marrow reserve. Liver and kidney functions were required to be within clinically acceptable ranges for this patient population: bilirubin <1.5 times higher than the normal value; aspartate aminotransferase and alanine transaminase <3 times higher than the normal range (both could be 5 times higher than normal in patients with known hepatic metastasis); and creatinine <1.5 times higher than the normal value. Chemotherapy was administered only via the intravenous route or via intravenous in combination with oral administration.

### Exclusion criteria

Pregnant or breast feeding women were excluded from participation in the study. Patients with previous bone marrow or stem cell transplantation or patients in whom high-dose chemotherapy or bone marrow or stem cell transplantation was anticipated in the 12 weeks following inclusion in the study were also excluded. Patients with an active infection or in receipt of an antibiotic in the 72 h before chemotherapy were also excluded, as were those with myeloid premalignant disease or malignant disease with myeloid characteristics, disease producing leukopenia, thrombocytopenia, or anaemia, and those who were receiving daily chemotherapy regimens or chemotherapy via a route other than intravenous or intravenous plus oral administration.

### Assessments

Each patient was assessed with regard to a number of demographic and disease-related variables that potentially influence chemotherapy-induced neutropenia. These included sex, body surface area, age-adjusted Charlson score, ECOG performance status score, baseline lymphocyte, neutrophil and platelet counts, baseline haemoglobin, bilirubin, creatinine, aspartate aminotransferase and alanine aminotransferase levels, treatment intention, and tumour-related variables (i.e. TNM stage). Neutropenia, defined as an absolute neutrophil count of <1 × 10^9^/L (Grade 3) or absolute neutrophil count <0.5 × 10^9^/L (Grade 4), was the main outcome variable studied. Febrile neutropenia was defined as severe neutropenia (Grade 4) with a body temperature of >38°C (Lyman *et al.* 2005). The blood test results were collected before each chemotherapy cycle, together with other tests requested by the physicians between the first and third chemotherapy cycles. No planned blood cell counts were performed at the cycle nadir.

### Statistical analysis

Descriptive analyses were used to construct the population profile and a variety of bivariate tests were performed to explore potential relationships between the independent pools of socio-demographic and clinical variables and chemotherapy-induced neutropenia in the population. A prediction tool was developed using a modelling process that incorporated clinically relevant second-order interactions between the variables. The hierarchical principle was applied when building the logistic regression model to allow result replication. This principle, applied to model building, states that when the interaction of two factors is significant, the individual factors must remain in the model (even if one or both factors are not statistically significant). The results are presented as odds ratios and 95% confidence intervals for variables in the chemotherapy-induced neutropenia prediction model, to provide information about their effect measure and its precision. The forward stepwise (Wald) decision process was used to determine the variables in the final model, and the input and output probability criteria for the variables were 0.05 and 0.1 respectively. A receiver operating characteristic curve was used to determine the cut-off point at which the sensitivity and specificity values for the model were maximised. All statistical operations were performed using the SPSS statistical software package, v12.

## RESULTS

Patients were enrolled and followed up between March 2004 and May 2006. A total of 1194 patients from 88 different oncology centres were enrolled. Their mean age was 58 ± 12 (SD) years, and 56% were women. Ninety-four per cent of the patients had a good initial performance status (ECOG 0–1). Breast (38%), lung (18%) and colorectal (15%) cancers were the most frequent cancers. Treatment intention was primarily radical, adjuvant or palliative. The most frequent chemotherapy regimens were platinum or anthracycline based. A minority of patients received concomitant radiotherapy (see Table 1). The incidence of grades 3–4 neutropenia was 10% (confidence interval 8.3–11.8; *n*= 116) during the first cycle of chemotherapy in our study population.

**Table 1:** Baseline characteristics of patients with solid tumours included in the study

	%	*n*
Demographics		
Age (years) (±SD)	58 ± 12	1194
Sex		
Female	56	1194
Male	44	
Clinical features		
ECOG performance score		
Score 0	62	1194
Score 1	32	
Score 2	6	
Tumour type		
Breast	38	1194
Trachea, bronchi and/or lungs	18	
Colon and rectum	15	
Ovary	5	
Stomach	5	
Other tumours (incidence <3%)	20	
Treatment intention		
Palliative	28	1194
Radical curative	9	
Radical neo-adjuvant	12	
Radical adjuvant	51	
Chemotherapy regimen		
Platinum-based chemotherapy	29	1194
Taxane-based chemotherapy	9	
Taxane + platinum-based chemotherapy	14	
Anthracycline-based chemotherapy	30	
Other	18	
Radiotherapy		
None	61	1194
Sequential	30	
Concomitant	9	
Laboratory values	mean ± SD	*n*
Haemoglobin (g/dL)	12.9 ± 1.6	1190
Lymphocytes (10^9^/L)	2.1 ± 1.8	1190
Neutrophils (10^9^/L)	5.3 ± 3.2	1190
Platelets (10^9^/L)	299.5 ± 110.9	1190
Bilirubin (mg/dL)	0.5 ± 0.5	1151
Aspartate aminotransferase (IU/L)	23.2 ± 15.7	1175
Alanine transaminase (IU/L)	25.5 ± 20	1184
Creatinine (mg/dL)	1.1 ± 4.0	1189

ECOG, Eastern Cooperative Oncology Group; SD, standard deviation.

### Model development

From all the demographic and disease characteristics assessed, baseline lymphocyte and neutrophil counts, ECOG performance status and the interaction between sex and treatment intention were found to influence chemotherapy-induced neutropenia and were included in the predictive model (see Table 2). The overall model significance was *P* < 0.0005.

**Table 2:** Multivariate logistic regression model predicting chemotherapy-induced neutropenia in patients with solid tumours initiating chemotherapy*

				95% confidence interval	
	Beta-coefficient	*P*-value	Odds ratio	Lower	Upper
Sex: male vs. female	−0.586	0.164	0.557	0.244	1.269
Baseline lymphocyte count	−0.400	0.011	0.670	0.492	0.913
Treatment intention		0.155			
Radical adjuvant vs. palliative	−0.434	0.274	0.648	0.298	1.410
Radical neo-adjuvant vs. palliative	−2.178	0.035	0.113	0.015	0.858
Radical curative vs. palliative	−0.319	0.509	0.727	0.282	1.874
Sex and treatment intention		0.012			
Male and radical adjuvant	0.455	0.413	1.576	0.531	4.679
Male and radical neo-adjuvant	2.889	0.012	17.975	1.893	170.697
Male and radical curative	1.835	0.013	6.263	1.470	26.688
ECOG performance score		0.011			
1 vs. 0	0.072	0.770	1.075	0.662	1.747
2 vs. 0	1.139	0.003	3.123	1.459	6.683
Baseline neutrophil count	−0.104	0.026	0.901	0.823	0.987

* Model significance (*P*= 0.0005, χ^2^).

Model constant: beta-coefficient =−0.693, *P*= 0.139 and odds ratio = 0.500.

ECOG, Eastern Cooperative Oncology Group.

The baseline lymphocyte count was inversely related to the risk of chemotherapy-induced neutropenia in the first cycle (odds ratio, 0.67; 95% confidence interval, 0.49–0.91), as was the baseline neutrophil count (odds ratio, 0.90; 95% confidence interval, 0.82–0.99). Patients with an ECOG status of 2 at baseline had a higher risk of chemotherapy-induced neutropenia in the first cycle than did patients with an ECOG of 0 (odds ratio, 3.12; 95% confidence interval, 1.46–6.68), and patients with neo-adjuvant treatment intention had a lower risk of chemotherapy-induced neutropenia than those whose treatment intention was palliative (odds ratio, 0.11; 95% confidence interval, 0.02–0.86). The interaction between sex and treatment intention was also predictive of chemotherapy-induced neutropenia.

**Figure 1:**
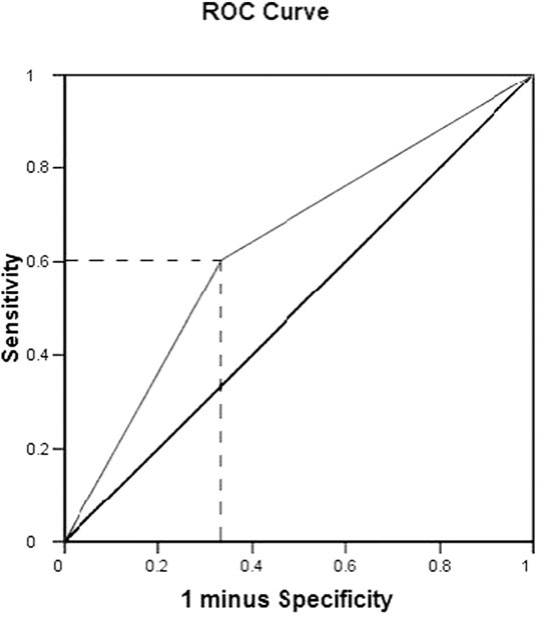
Determining model sensitivity and specificity with the receiver operating characteristic curve (ROC).

**Figure 2:**
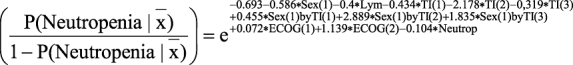
Logistic regression model equation for the prediction of first-cycle chemotherapy-induced neutropenia. Sex (1), male; Lym, baseline lymphocyte count; TI (1), treatment intention: radical adjuvant; TI (2), treatment intention: radical neo-adjuvant; TI (3), treatment intention: radical curative; ECOG (1), 1; ECOG (2), 2; Neutrop, baseline neutrophil count. ECOG, Eastern Cooperative Oncology Group.

A cut-off point of 0.101 on the receiver operating characteristic curve provided maximal values for sensitivity (63%) and specificity (67%). This cut-off point maximises the true positives and the true negatives of the equation (see Fig. 1 and Table 3). Using the model equation in Figure 2, results ≥0.101 predicted first-cycle chemotherapy-induced neutropenia, whereas results <0.101 predicted those patients in whom no chemotherapy-induced neutropenia would occur. In our study population, 36% of patients (*n*= 430) were identified as at high risk of first-cycle chemotherapy-induced neutropenia and 64% (*n*= 764) as at low risk of grade 3 or 4 neutropenia. The positive predictive value (i.e. patients having neutropenia when the model predicted it) was 17% (95% confidence interval, 13.6–21.4), and the negative predictive value (i.e. patients who did not develop neutropenia when the model predicted no neutropenia) was 94% (95% confidence interval, 92.2–95.9). The sensitivity and specificity of the predictive risk model for first-cycle chemotherapy-induced neutropenia were 63% and 67% respectively.

**Table 3:** Discriminatory properties of the multivariate logistic regression model predicting chemotherapy-induced neutropenia

	Expected by the model		
	Chemotherapy-induced neutropenia		
Observed in the study (gold standard)	No	Yes	
Chemotherapy-induced neutropenia			
No	654	321	975 (specificity: 67%)
Yes	40	67	107 (sensitivity: 63%)
	694 (negative predictive value: 94%)	388 (positive predictive value: 17%)	1082 (overall correct: 67%)

## DISCUSSION

Two basic types of models have been developed to predict neutropenia, which can be found in the literature: conditional models, which are based on patient toxicity during the first chemotherapy cycle, and unconditional models, which are dependent on pretreatment values (Crawford *et al.* 2004; Lyman *et al.* 2005). Our model is of the latter type, and aims to predict (and therefore help to avoid) the first episode of neutropenia. Neutropenic complications are most likely to occur in the first cycle of chemotherapy, so unconditional models are more appropriate.

The resulting logistic regression model was based on four factors: (1) baseline neutrophil; (2) lymphocyte counts, which were, as expected, inversely related to neutropenia because of their relationship with the patient's bone marrow reserve (these factors have been found to be useful in other risk models) (Silber *et al.* 1998; Rivera *et al.* 2003); (3) ECOG scores: higher ECOG scores were associated with increased toxicity; and (4) the interaction of sex and treatment intention (the latter two factors were included because their interaction was significant). This interaction suggests different patterns of chemotherapy-induced neutropenia risk according to treatment intention in men and women, and may also be a function of the large breast cancer subgroup in the study, which constituted over one-third of the total study population.

The incidence of chemotherapy-induced neutropenia in the study population was 10%. With a positive predictive value of 17%, the use of the model actually increases by 7% of the power to predict neutropenia in approximately one-third of our population (i.e. those for whom the model predicts neutropenia). The model also increases to 94% of the power to predict no neutropenia in the remainder (64%) of the population (i.e. those for whom the model predicts no risk of neutropenia). Therefore, in two-thirds of the patients, a negative prediction could be useful in reducing the chance of suffering neutropenia from 10% to 6% in the first cycle. In a practical sense, the use of this model will allow physicians to circumscribe their efforts (closer follow-up and/or primary prophylaxis) to a smaller group of patients at higher risk of developing chemotherapy-induced neutropenia, while selecting out a larger group of patient with no risk of chemotherapy-induced neutropenia. To examine the model more closely, we applied it to a population in which the incidence of chemotherapy-induced neutropenia was about 20%, and a positive predictive value of 32% was obtained (data not shown), which represents an increase of 12% in the predictive power of the model (as the sensitivity and specificity of the model will not change in a real population).

As previously mentioned, other risk models have identified several risk factors for the development of chemotherapy-induced neutropenia/febrile neutropenia and its related mortality. These models and risk factors are disease specific (e.g. breast cancer, lung cancer, leukaemia) or regimen oriented. Our model was developed in a large adult population with diverse cancers and the data were collected with the aim of identifying haematological toxicities in general oncology practice. Therefore, we believe that the present model represents a step forward, providing a useful tool for the prediction of neutropenia in normal oncology practice.

Two factors that have commonly been included in previous models were not included in our model: age and chemotherapy regimen. Our justification for the omission of age is that this factor showed collinearity with a number of the other variables (such as lymphocyte count, co-morbidity, ECOG status, chemotherapy, treatment intention) during the modelling process. When age was included, many other factors became insignificant, indicating that age is a particularly strong predictive factor for neutropenia, and thus excludes other variables from the equation. Age is already a known risk factor for the development of chemotherapy-induced neutropenia/febrile neutropenia. Therefore, its omission is reasonable. Furthermore, chronological age and biological age can differ, and the results of some studies have suggested that the measure of clinical frailty could be a risk factor rather than age per se (Crawford *et al.* 2004). Our modelling process for the equation seems to corroborate this, because the ECOG functional status measure was identified as a significant factor, although in our sample, the majority of patients were only ECOG 0–1, with 6% of patients ECOG 2, and none with higher values because of the study design. Our main reasons for not including the chemotherapy regimen in the model are the diversity of regimens used for the treatment of non-specific cancers and the evolution of those treatments as new drugs are incorporated. Instead, we included treatment intention, which potentially reflects the chemotherapy dose administered. This model has only been assessed against itself, so it must be validated before its use in a clinical setting.

The large number of patients and the quality of the data collected in the DELFOS study, the prospective design, together with the diversity of patients and solid tumours included in the study give this model a sound basis. However, we acknowledge that it is difficult to develop a single model that fits the diversity of patient characteristics, diseases and chemotherapy regimens observed in our sample. Future studies will focus on the validation of the model in general and in specific oncological populations, and also on the evaluation of its impact on morbidity, mortality and economics.

## CONCLUSION

This risk assessment model allows the identification of high- and low-risk patients before the initiation of chemotherapy. A prospective study must be performed to validate it. The model represents a step forward in chemotherapy-induced neutropenia risk management.
